# IGFBP7 induces phenotypic shift from platelet-mediated autoagglutination to rosetting of *Plasmodium knowlesi*-infected erythrocytes

**DOI:** 10.1080/21505594.2026.2711530

**Published:** 2026-08-27

**Authors:** Joo-Yie Chin, Laurent Rénia, Wenn-Chyau Lee

**Affiliations:** a Department of Parasitology, Faculty of Medicine, Universiti Malaya, Kuala Lumpur, Malaysia; b Lee Kong Chian School of Medicine, Nanyang Technological University (NTU), Singapore, Singapore; c School of Biological Sciences, Nanyang Technological University (NTU), Singapore, Singapore; d A*STAR Infectious Diseases Labs (A*IDL), Agency for Science, Technology and Research (A*STAR), Singapore, Singapore

**Keywords:** Malaria, autoagglutination, rosetting, plasmodium knowlesi, plasmodium falciparum, IGFBP7

## Abstract

Zoonotic malaria caused by *Plasmodium knowlesi* is an emerging infectious disease with persistent transmission in Southeast Asia over the past two decades. This zoonosis causes potentially life-threatening hematological complications such as thrombocytopenia. While mechanisms behind the malaria-induced acute thrombocytopenia remain to be fully deciphered, it is believed to be associated with the cytoadherence of *Plasmodium*-infected erythrocytes (IRBC) to platelets. Of note, platelet-mediated cytoadherence of IRBC gives rise to platelet-mediated clumping of IRBC, also known as IRBC autoagglutination. Nevertheless, most of the autoagglutination studies were performed with *P. falciparum*. In this study, we characterized autoagglutination of *P. knowlesi* and *P. falciparum*, and investigated the relationship between IRBC autoagglutination and rosetting rates (another IRBC cytoadherence phenomenon). Autoagglutination was found to be dependent on the presence of human serum and platelets, where the presence of platelets favored IRBC autoagglutination over rosetting. The autoagglutination of *P. knowlesi* and *P. falciparum* was mediated by CD36, where antibody blockade of CD36 inhibited autoagglutination and drove the IRBC to form more rosettes with the uninfected erythrocytes (URBC). Antibody blocking of P-selectin reduced only the autoagglutination of *P. falciparum* with no effect on the rosetting rates. Notably, the presence of IGFBP7 (a human-derived protein that facilitates the formation of type II rosettes) suppressed autoagglutination while stimulating rosetting rates of IRBC. Given the ability of IGFBP7 to alter the *Plasmodium*-mediated cytoadherence dynamics between autoagglutination and rosetting, this human protein may alter the pathobiology of malaria, hence the disease outcomes of the infection.

## Introduction

The transmission of zoonotic malaria caused by *Plasmodium knowlesi* has contributed significant healthcare burden to Southeast Asia [1,2], and presented different challenges of malaria elimination to the affected nations [3]. Unlike many other zoonoses that cause asymptomatic infections or mild symptoms [4], knowlesi malaria is potentially fatal, and cases with severe complications have been reported [5–7]. One of the most reported complications among knowlesi malaria patients is thrombocytopenia, encountered even in cases with low parasitemia [6–8]. The mechanisms behind knowlesi malaria-induced thrombocytopenia have yet to be fully deciphered. Nevertheless, the interactions between infected erythrocytes (IRBC) and platelets have been demonstrated to play crucial roles in the immuno-pathobiology of malaria, including infections caused by *P. knowlesi* [7,9–12]. Indeed, platelets mediate the autoagglutination of IRBC (stable clumping of several IRBC) [13,14], which is an IRBC-derived cytoadherence phenomenon distinct from rosetting [stable binding of IRBC to uninfected erythrocytes (URBC)], another similar-looking IRBC cytoadherence phenomenon within the circulation [15]. Notably, rosetting phenomenon can be mediated by Insulin-like Growth Factor-Binding Protein 7 (IGFBP7) via a complex interaction with thrombospondin-1 (TSP-1) and Von Willebrand Factor (VWF) [16], which are crucial players that mediate platelet adhesion [17,18]. The relationship between rosetting and autoagglutination in malaria pathogenesis remained elusive.

An earlier study described agglutination of simian *P. knowlesi*-IRBC exposed to immune sera from monkeys that were subjected to chronic *P. knowlesi* infection, where the effect was parasite species-specific and not found with sera of monkeys that experienced acute *P. knowlesi* infection [19]. However, this differs from autoagglutination which is antibody-independent but requires platelets as shown for *P. falciparum* [20]. Binding of clinical isolate-derived *P. knowlesi*-IRBC to platelets has been demonstrated [9]. Despite the increasing research attention on the cytoadherence of human *P. knowlesi-*IRBC [21–25], *P. knowlesi*-IRBC autoagglutination remained to be fully characterized. In this study, we investigated IRBC autoagglutination by *P. knowlesi* and *P. falciparum*. We also determined the effect of IGFBP7 on the equilibrium of rosetting and IRBC autoagglutination by *P. knowlesi* and *P. falciparum*.

## Materials and methods

### Ethical considerations

The study was conducted in accordance with the Declaration of Helsinki, along with ethical approval and written informed consent by the Institutional Biosafety and Biosecurity Committee of Universiti Malaya (UMIBBC/PA/R/FOM/PARA-025/2022) and Medical Research Ethics Committee of Universiti Malaya Medical Center (MRECID.No 2,024,312-13526).

### General experiment conditions

Each experiment was conducted with eight biological replicates (distinct parasite culture batches), unless stated otherwise. All replicates represented independent parasite cultures derived from different culture batches. All experiments were performed at 3% parasitemia and 2% hematocrit unless otherwise specified. Experiments were conducted with parasite culture suspension demonstrating at least 70% of the parasite population being late asexual erythrocytic stages (late trophozoite – schizont stages). Information regarding the materials and reagents used in this study is available as Supplementary Table S1.

### IGFBP7 protein suspension preparation

Recombinant human IGFBP7 was reconstituted (100 µg/mL) with 1X PBS by following the manufacturer’s instructions. The protein suspension stocks were stored at −20°C until further use.

### Parasite culture

*P. knowlesi* A1-H.1 and *P. falciparum* 3D7 cultures were maintained at 2% hematocrit and 3% parasitemia. The parasites were propagated with human RBC (from healthy uninfected individual adults following the informed consent) in RPMI 1640 medium supplemented with 0.5% AlbuMAX II and 10% heat-inactivated human serum (pooled group B sera from healthy uninfected individual adults following the informed consent), and maintained at 37°C, with relative humidity (RH) of >90%, and gas conditions of 5% CO_2_, 5% O_2_, as described elsewhere [16,26–28]. Late-stage parasites were isolated using a Magnetic Activated Cell Sorter (MACS) for cytoadherence experiments [28], which used the matched serum-enriched media.

Human whole blood from healthy uninfected individual adults was collected into sodium citrate vacutainers (following the informed consent) and processed according to the protocols described elsewhere [13]. Briefly, whole blood was centrifuged at 250 g for 10 minutes to obtain platelet-rich plasma (PRP). The PRP was then carefully aspirated without disturbing the RBC layer and transferred to another centrifuge tube. Platelets were isolated from the PRP through additional centrifugation at 800 g for 15 minutes. The platelets were sedimented, whereas the supernatant containing platelet-poor plasma (PPP) was discarded. The isolated platelets were resuspended gently in 1X PBS and kept at 4°C until further use for experiments within the same day, with their concentration determined using the cell counting chamber.

### Rosetting assay

The Giemsa-stained wet mount technique was employed to determine the rosetting rate [29,30]. Briefly, the parasite culture suspension was stained with a 5% Giemsa subvital working solution for 15 minutes at room temperature before preparing wet mounts for the rosetting assay. The slides were examined under a light microscope at 1000X magnification. Rosetting (defined as an IRBC stably bound to at least two URBC) rate was assessed by recruiting 200 IRBC.

$$\begin{array}{rl} & \mathrm{Rosetting}\;\;\mathrm{rate}\left(\%\right)\\ & \;\;=\frac{\mathrm{number}\;\;\mathrm{of}\;\mathrm{iRBC}\;\;\mathrm{formed}\;\;\mathrm{rosette}}{200\;\;\mathrm{iRBC}\;\mathrm{recruited}}\times 100\% \end{array}$$

### IRBC autoagglutination assay

Autoagglutinated IRBC were quantified using the same wet mount method as the rosetting assay described above. The autoagglutination rate, which indicates the percentage of aggregated IRBC without the involvement of URBC, out of the 500 recruited IRBC, was determined [14,25,31].

$$\begin{array}{rl} & \mathrm{Autoagglutination}\;\;\mathrm{rate}\left(\%\right)\\ & \;\;\;\;\;\;\;\;\;\;\;\;=\frac{\mathrm{agglutinated}\;\;\mathrm{IRBCs}}{500\;\;\mathrm{IRBCs}\;\;\mathrm{recruited}}\times 100\% \end{array}$$

The IRBC autoagglutination and rosetting rates were evaluated as mutually exclusive phenotypic categories. Quantitative tracking across all experimental fields confirmed that individual IRBC did not simultaneously exhibit both phenotypes. Phenotypic scoring was executed using a semi-blinded design: independent, trained assessors quantified the slides without knowledge of the experiment treatment groups. Assessors could not be blinded to the parasite species due to detectable morphological differences between late-stage *P. falciparum* and *P. knowlesi*. The fluidic setting of wet mount helped omitting random cell overlaps. Inter-assessor consistency was verified prior to data collection [intra-class correlation coefficient (ICC) >0.90].

### Effect of platelets and human serum on IRBC autoagglutination and rosetting

The packed RBC derived from parasite culture were divided into two groups. One group was incubated in 10% human serum-enriched medium with 1.5 × 10^6^ platelets, while the other served as the platelet-free control. The parasite suspension was then incubated under standard culture conditions for two hours before assessing rosetting and autoagglutination. Both rosetting and autoagglutination rates counting were performed concurrently. The experiment was repeated with media of different levels of human serum enrichment (0% and 20% vol/vol).

### Characterization of receptors involved in IRBC autoagglutination

The IRBC autoagglutination assay was repeated with slight modifications, where one group was added with 10 µg/ml rabbit anti-human CD36 IgG, the second group was added with 10 µg/ml rabbit anti-human P-selectin IgG, and another group was added with 10 µg/ml rabbit anti-human PECAM-1 (CD31) IgG. For control experiments, a separate group was added with 10 µg/ml matched normal rabbit IgG isotype control, and another group served as antibody-free control. Following two hours of parallel incubation under *in vitro* cultivation conditions for all experiment and control groups, autoagglutination and rosetting assays were performed.

### Effect of IGFBP7 on equilibrium of IRBC autoagglutination and rosetting

The purified late-stage IRBC pellet was divided into four groups, to be subjected to the following experimental settings: platelet-free and IGFBP7-free (control; i.e. no platelets, no IGFBP7), platelet-free with 100 ng/mL IGFBP7, 1.5 × 10^6^ platelets without IGFBP7, and 1.5 × 10^6^ platelets with 100 ng/mL IGFBP7. Autoagglutination and rosetting assays were conducted after two hours of incubation under *in vitro* cultivation conditions. The equilibrium between IRBC autoagglutination and rosetting was operationally defined as the relative proportions of IRBC engaged in each mutually exclusive phenotype under a given condition. Of note, 100 ng/ml IGFBP7 was selected, based on the rosetting rate profiling of the laboratory-adapted malaria parasites used in this study under different IGFBP7 working concentrations (Supplementary Figure 1).

### Statistical analyses

GraphPad Prism 10.6 was used for data analyses. Data normality was evaluated with Shapiro-Wilk test. For comparison of multiple groups of normally distributed data with unequal variances, Welch’s ANOVA with Dunnett’s T3 test was conducted. To compare multiple groups with multiple independent variables, three-way ANOVA with Tukey’s test was performed. Simple linear regression was used to evaluate the nature of relationship between two parameters of interest. *p* values smaller than 0.05 were considered as statistically significant in the analyses.

## Results

### Platelets and human serum are essential for IRBC autoagglutination

**Figure 1.**
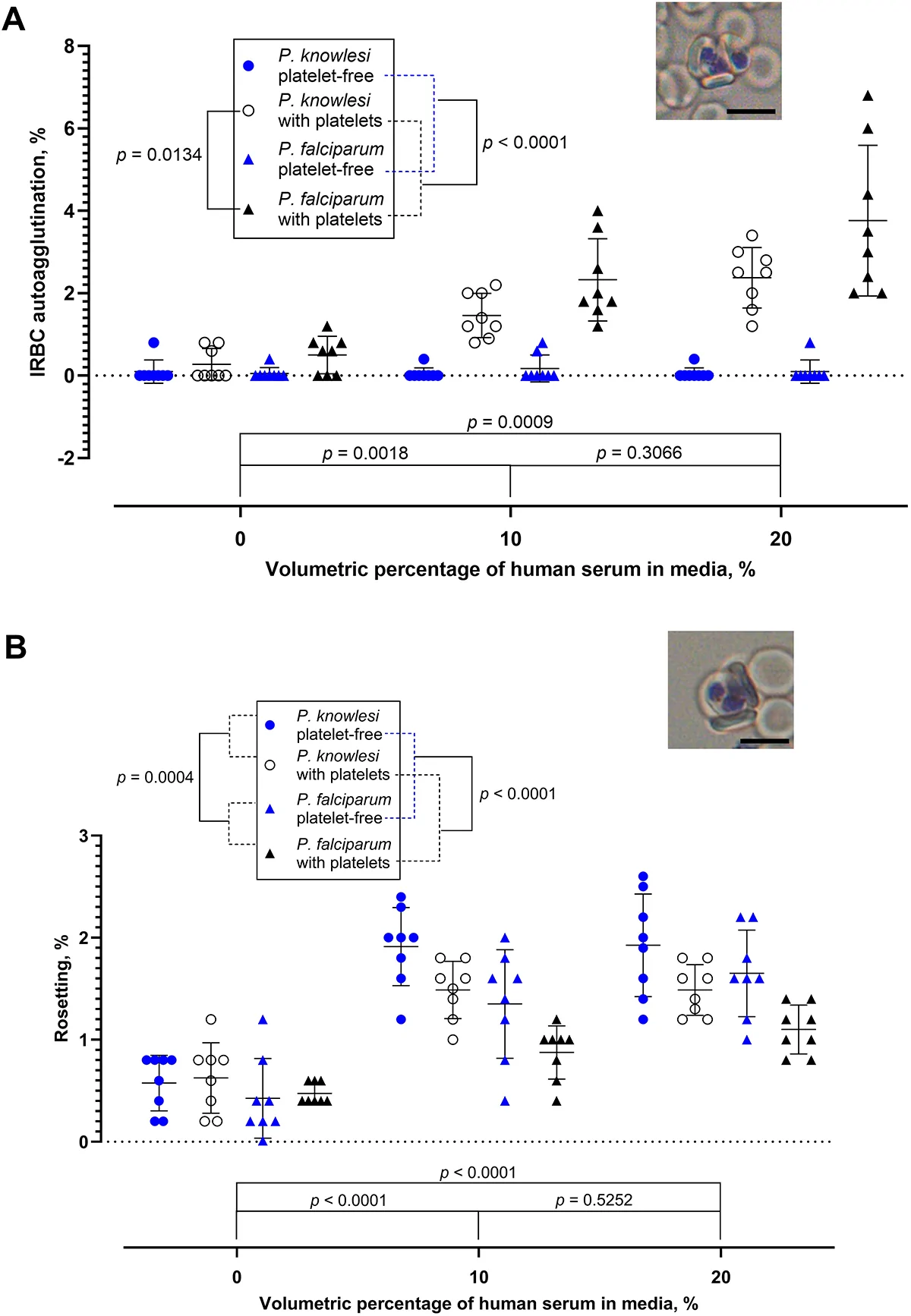
Effects of platelets and human serum on IRBC autoagglutination (A) and rosetting (B) of *P. knowlesi* and *P. falciparum*, with photo insets showing the IRBC engaged in respective phenomena. Three-way ANOVA with Tukey’s multiple comparison test was performed to analyze the data [IRBC autoagglutination (Supplementary Table S2) and rosetting (Supplementary Table 3)]. Error bars represent mean and SD (values available in data repository). Scale bar in photo insets: 10 µm.

The IRBC autoagglutination rates were significantly dependent upon the availability of platelets (*p* < 0.0001) and human serum (*p* < 0.0001), with *P. falciparum* demonstrating higher autoagglutination rates than *P. knowlesi* (*p* = 0.0134) (Figure 1(A)). Of note, there was no significant difference between IRBC autoagglutination rates in 10% serum-containing medium and those observed in 20% serum containing medium. Between both species tested, there was no significant difference with regards to the impact of platelets and serum availability to IRBC autoagglutination (Supplementary Table S2).

The rosetting rates were analyzed in parallel. They were significantly influenced by the presence of human serum (*p* < 0.0001) and the availability of platelets (*p* < 0.0001), where the presence of platelets under serum-supplied conditions lowered the rosetting rates (Figure 1(B)). No significant difference was found between rosetting rates recorded in 10% serum-supplied setting and those recorded in 20% serum-supplied condition (Supplementary Table 3).

**Figure 2.**
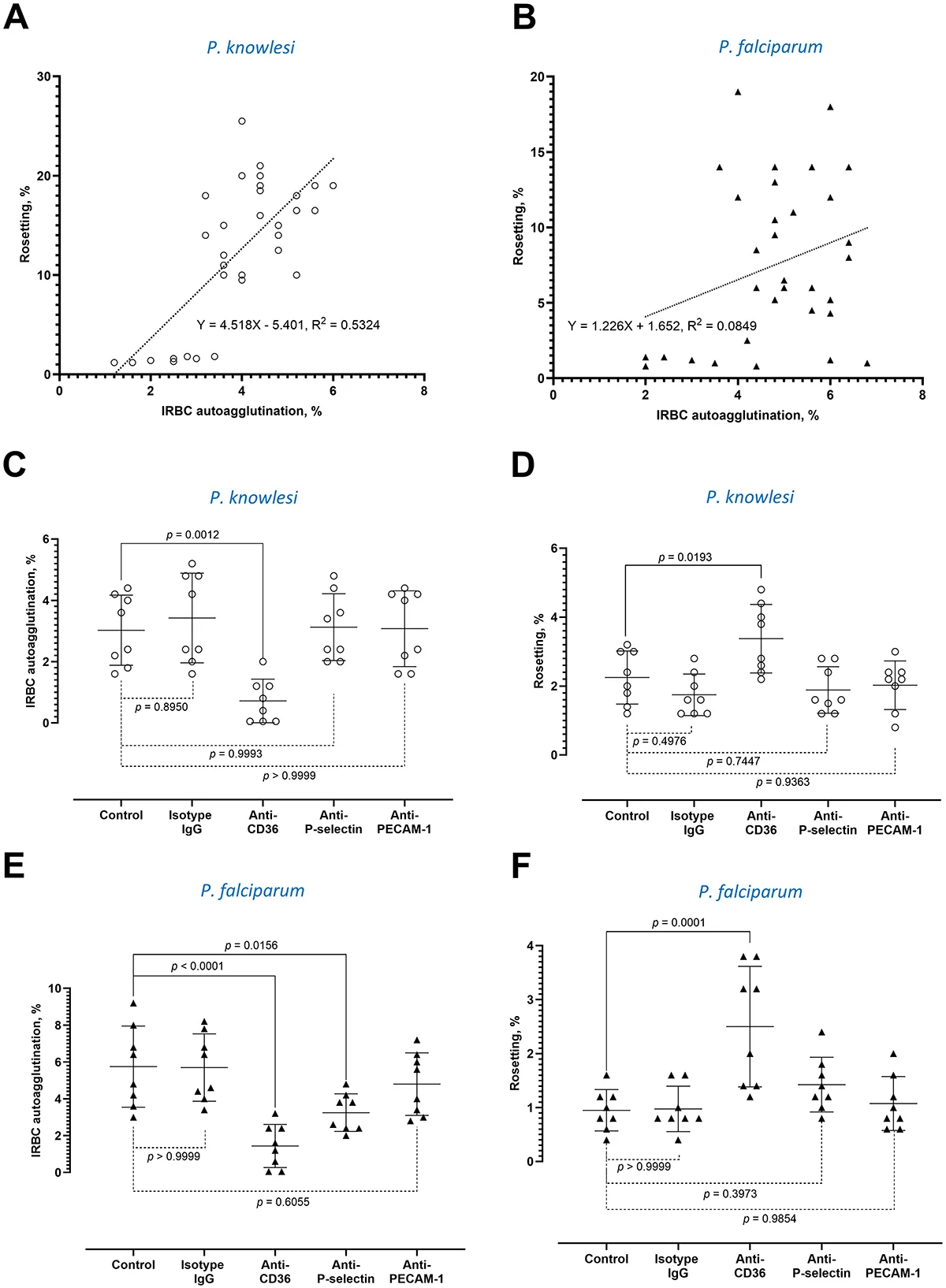
Further characterization of IRBC autoagglutination and rosetting phenomena by *P. knowlesi* and *P. falciparum*. Simple linear regression of IRBC autoagglutination and rosetting for *P. knowlesi* (F_1,30_ = 34.16; *p* < 0.0001; R^2^ = 0.5324) (A) and *P. falciparum* (F_1,30_ = 2.785; *p* = 0.1056; R^2^ = 0.0849) (B); 32 biological replicates were recruited for each species. Effect of antibody blocking of CD36, P-selectin and PECAM-1 on IRBC autoagglutination (one-way ANOVA F_4,35_ = 7.301, *p* = 0.0002) (C) and the concomitant rosetting rates (one-way ANOVA F_4,35_ = 5.865; *p* = 0.0010) (D) by *P. knowlesi*-IRBC. Effect of antibody blocking of CD36, P-selectin and PECAM-1 on IRBC autoagglutination (one-way ANOVA F_4,35_ = 10.02; *p* < 0.0001) (E) and the concomitant rosetting rates (one-way ANOVA F_4,35_ = 8.184; *p* < 0.0001) (F) by *P. falciparum*-IRBC. Linear regression analysis was performed utilizing an expanded dataset of *n* = 32 historical data points compiled across continuous culture maintenance to ensure statistical power, whereas individual comparative assays utilized *n* = 8 strictly independent biological replicates. Error bars represent mean and SD (values available in data repository).

A simple linear regression was performed to evaluate if IRBC autoagglutination rates predict the IRBC rosetting rates of *P. knowlesi* and *P. falciparum*. For *P. knowlesi*, they were significantly, positively correlated (Figure 2(A)). On the other hand, for *P. falciparum*, there was no significant correlation between rosetting and autoagglutination (Figure 2(B)).

### Receptors involved in IRBC autoagglutination of P. knowlesi and P. falciparum

Anti-CD36 antibodies significantly hampered the IRBC autoagglutination of *P. knowlesi* (*p* = 0.0012) (Figure 2(C)) and concomitantly led to an increment in the rosetting rates (*p* = 0.0193) (Figure 2(D)). Antibody blocking of P-selectin and PECAM-1 did not significantly alter the IRBC autoagglutination and rosetting rates of *P. knowlesi*. For *P. falciparum*, antibody blocking of CD36 and P-selectin significantly reduced the IRBC autoagglutination (*p* < 0.0001 for anti-CD36, and *p* = 0.0156 for anti-P-selectin) (Figure 2(E)). Of note, addition of anti-CD36 antibodies resulted in a concomitant increase in *P. falciparum* rosetting rates (*p* = 0.0001). However, no significant changes in *P. falciparum* rosetting rates were observed after the addition of anti P-selectin antibodies (Figure 2(F)).

### IGFBP7 altered the IRBC cytoadherence equilibrium between autoagglutination and rosetting

**Figure 3.**
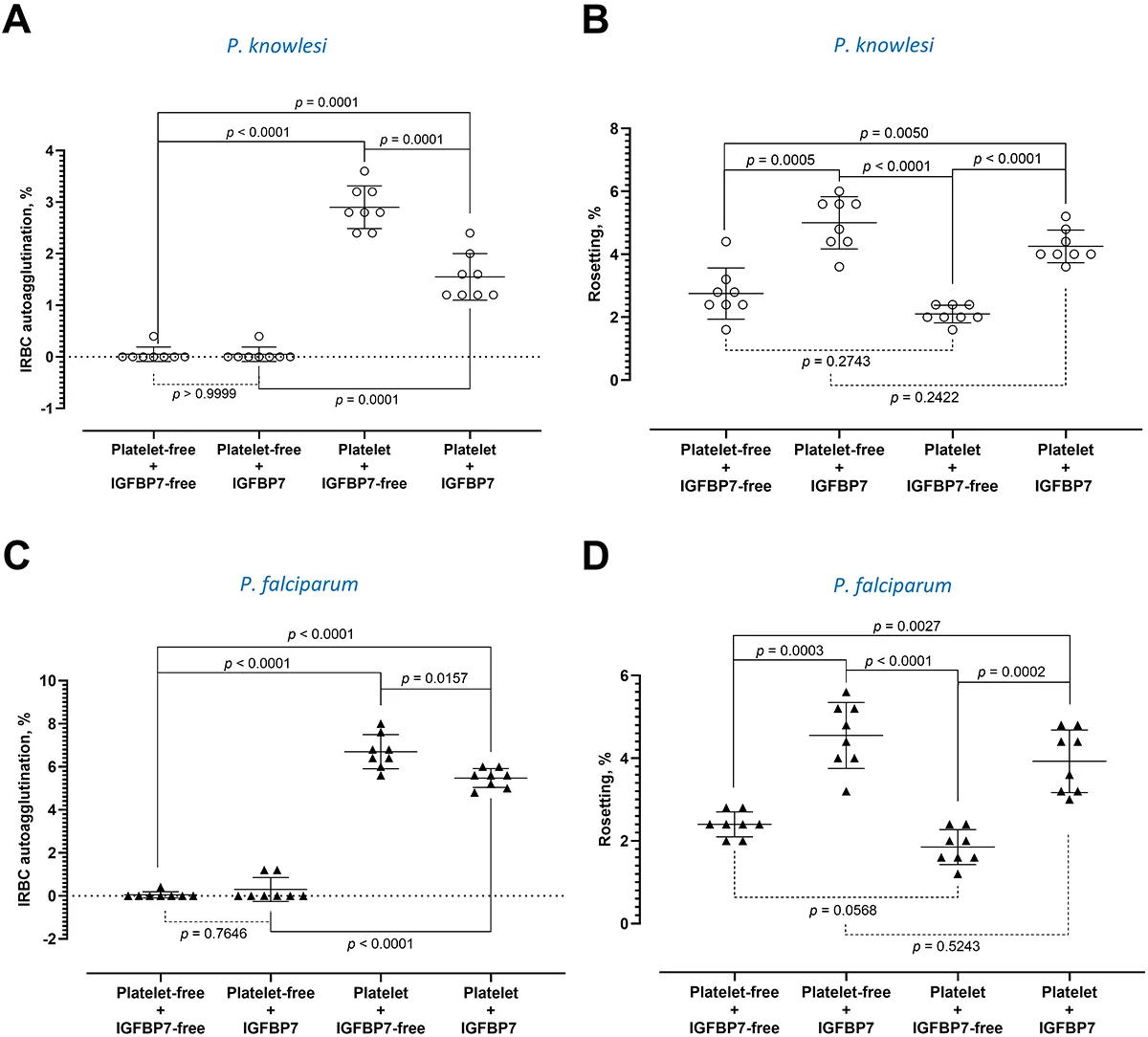
Effect of IGFBP7 on the equilibrium between IRBC autoagglutination and rosetting. Data were analyzed using Welch’s ANOVA. Changes on the IRBC autoagglutination (Welch’s ANOVA W_3,13.94_ = 148.5; *p* < 0.0001) (A) and the concomitant rosetting rates (Welch’s ANOVA W_3,14.14_ = 51; *p* < 0.0001) (B) of *P. knowlesi* following exposure to IGFBP7. Effect of IGFBP7 on IRBC autoagglutination (Welch’s ANOVA W_3,11.67_ = 542.2; *p* < 0.0001) (C) and the concomitant rosetting rates (Welch’s ANOVA W_3,14.60_ = 30.69; *p* < 0.0001) (D) by *P. falciparum*-IRBC. Error bars represent mean and SD (values available in data repository).

Addition of IGFBP7 to the cultures significantly altered the dynamics of *P. knowlesi*- IRBC autoagglutination (*p* = 0.0001) (Figure 3(A)). Co-incubation of parasite culture suspension with platelets and IGFBP7 reduced *P. knowlesi*-IRBC autoagglutination, as compared to that of parasite culture suspension incubated with platelets under IGFBP7-free condition (*p* = 0.0001). The presence of IGFBP7 altered the rosetting rates of *P. knowlesi* significantly (*p* < 0.0001) (Figure 3(B)), where the IGFBP7-mediated rosette-stimulation was observed in platelet-free setting (*p* = 0.0005) and the platelet-supplied condition (*p* < 0.0001). There was no significant difference in the IGFBP7-mediated rosetting between platelet-free and platelet-supplied settings (*p* = 0.2422).

Likewise for *P. falciparum*, IGFBP7-mediated reduction of IRBC autoagglutination was observed (*p* < 0.0001) (Figure 3(C)), with a simultaneous increase in rosetting rates (*p* < 0.0001) (Figure 3(D)). The IGFBP7-mediated rosette-stimulation was not affected by the presence of platelets (*p* = 0.5243).

## Discussion

Here in this study, we described for the first time, the platelet-mediated autoagglutination of *P. knowlesi*-IRBC. Similar to *P. falciparum*, the IRBC autoagglutination for *P. knowlesi* is platelet-dependent, and requires serum-derived components [13,14]. During malaria pathogenesis, platelets are consumed to facilitate the clearance of malaria parasites by the host’s innate immune system [9,32]. IRBC agglutination may further aggravate the consumption and exhaustion of platelets during the infection, leading to the acute onset of thrombocytopenia. Indeed, malaria-associated thrombocytopenia is reversed rapidly following parasite clearance by successful antimalarial treatment [33,34], suggesting that malaria-associated thrombocytopenia is more likely resulting from the IRBC-associated exhaustion of host’s platelet reserve, instead of compromised platelet synthesis.

We uncovered a significant relationship between IRBC autoagglutination and the rosetting phenomenon for *P. knowlesi*, which was not observed for *P. falciparum*. To date, several parasite-derived proteins have been incriminated as the cytoadherence ligands in different cytoadherence events by *P. falciparum*-IRBC, namely PfEMP1, STEVOR, RIFIN, and PfGARP [15]. Among these, PfEMP1 mediates the binding of *P. falciparum-*IRBC to endothelial cells, leukocytes, syncytiotrophoblast, URBC (rosetting), and IRBC (autoagglutination) [12,15]. The binding targets for STEVOR, RIFIN and PfGARP are URBC [35–37], with RIFIN being demonstrated to interact with leukocyte-derived receptors as well [38]. The single ligand-mediated autoagglutination and multiple ligands-mediated rosetting may contribute toward the lack of significant relationship between the two phenomena in *P. falciparum*. For *P. knowlesi*, the cytoadherence interactions of IRBC with different host-derived receptors are primarily mediated by the SICA proteins [20,39]. This may explain the significant relationship between rosetting and autoagglutination of *P. knowlesi*-IRBC.

The role of CD36 in IRBC autoagglutination by *P. knowlesi* and *P. falciparum* was demonstrated by the antibody blocking assay, which was accompanied by a concomitant increase of rosetting rates in both species. This suggests that IRBC autoagglutination and rosetting compete for shared parasite cytoadherence ligands. When IRBC autoagglutination is blocked by antibody, the unoccupied ligands may mediate rosetting with URBC. Alternatively, the binding domains correspond to autoagglutination and rosetting may be adjacent to each other on the same parasite ligand. When the stearic hindrance against rosetting is lifted (due to the antibody blockade of autoagglutination), the rosette-mediating domain is allowed to interact with non-CD 36 receptors on URBC for rosette formation. This may also explain why we did not encounter any single IRBC committing to rosetting and autoagglutination simultaneously. Previously, a role for CD36 was proposed in *P. falciparum* rosette formation [40–42]. Nevertheless, we observed a significantly higher rosetting rates for *P. falciparum* and *P. knowlesi* following incubation of IRBC and platelets with anti-CD36 antibody. This suggests that CD36 present on URBC is not involved in rosette formation by both species of malaria parasites. In fact, our findings agreed with another earlier study that demonstrated failure of anti-CD36 antibody to inhibit rosetting by *P. falciparum* [43].

Demonstrated only for *P. falciparum*, blocking of P-selectin inhibited IRBC autoagglutination without altering the rosetting rates. Of note, P-selectin is not expressed on RBC [44,45], and the interaction between PfEMP1 and P-selectin has been demonstrated to facilitate the interaction between *P. falciparum*-IRBC and CD36 on platelets and endothelial cells [46]. Based on our antibody blocking assays, the occurrence of *P. falciparum* rosetting is independent of the PfEMP1 or PfEMP1 domains that interact with P-selectin [47,48]. This may also contribute toward the lack of significant relationship between *P. falciparum* rosetting and autoagglutination. In short, *P. knowlesi*-IRBC autoagglutination is mediated by CD36, whereas *P. falciparum*-IRBC autoagglutination is mediated by both CD36 and P-selectin.

Finally, the presence of IGFBP7 was found to disrupt the binding equilibrium of IRBC, shifting the preference from autoagglutination toward rosetting in both *P. knowlesi* and *P. falciparum*. Notably, IGFBP7 stimulated rosetting even in platelet-free environment. This reflects that IGFBP7 exerts a direct stimulatory effect on the interactions between the IRBC and URBC, instead of merely acting as a passive inhibitor of platelet-mediated clumping. By prioritizing rosette formation with URBC over autoagglutination of IRBC, IGFBP7 may alter the dynamics of platelet consumption during active pathogenesis. However, the clinical consequences of this phenotypic shift remain ambiguous. While a reduction in autoagglutination could theoretically protect the host from severe thrombocytopenia by sparing the platelet reserve, rosetting has been associated with severe malaria in earlier studies [12]. Nevertheless, rosettes have been demonstrated to facilitate parasite sequestration in deep vasculature without completely occluding the affected vasculature [49]. Therefore, whether the net effect of an IGFBP7-mediated shift is protective or harmful to the host remains to be determined. While our data demonstrate a clear phenotypic shift mediated by IGFBP7, we acknowledge that direct competitive binding assays, platelet consumption assays, or *in vivo* animal studies were not performed. Besides, evaluating the circulating levels of IGFBP7 in malaria patients of different disease severity remains an important gap to fill. Of note, IGFBP7 interacts with thrombospondin 1 (TSP-1) and von Willebrand Factor (VWF) [16]. Uncovering the specific roles of these downstream molecular bridges represents another exciting avenue to decipher these interaction mechanisms. Further biochemical and functional characterization is essential to map this molecular competition, and evaluate whether modulating this equilibrium holds any clinical viability as an adjunct therapy for the management of malaria.

## Conclusion

The autoagglutination of *P. knowlesi*-IRBC is a platelet- and serum-dependent cytoadherence phenomenon that is mediated by CD36. This cytoadherence phenomenon is significantly related to the rosetting phenomenon of *P. knowlesi*. For both *P. knowlesi* and *P. falciparum*, the presence of IGFBP7 skewed the cytoadherence inclination of IRBC toward rosetting over autoagglutination, which may reduce the exhaustion of host platelets from being engaged in IRBC-mediated cytoadherence events.

## Supplementary Material

Supplementary materials_Chin et al_2026_revised.docx

## Data Availability

The authors confirm that the data and information supporting the findings of this study are available within the article and its supplementary material. The data that support the findings of this study are openly available in Universiti Malaya Research Data Repository (https://researchdata.um.edu.my/) at https://doi.org/10.22452/RD/NA9YBC, reference number [50].
