# Supplementary material for: IGFBP7 induces phenotypic shift from platelet-mediated autoagglutination to rosetting of Plasmodium knowlesi-infected erythrocytes
Source: Virulence. 2026 Aug 27;17(1):2711530. doi: 10.1080/21505594.2026.2711530 (PMC13531942; doi:10.1080/21505594.2026.2711530)
Supplement: Supplementary file 1 [file KVIR_A_2711530_SM0386.docx]

**Supplementary materials**

**Supplementary Table 1.	Information regarding the materials and reagents used in this study.**

| **Reagent/Resource** | **Reference or source** | **Identifier/ Catalog Number / Notes** |
| --- | --- | --- |
| **Biological materials** |  |  |
| A1-H.1 (*Plasmodium knowlesi*) | Laboratory-adapted parasite strain; maintained in Universiti Malaya | Used for all experiments |
| 3D7 (*Plasmodium falciparum*) | Laboratory-adapted parasite strain; maintained in Universiti Malaya and A*STAR ID labs. | Used for all experiments |
| **Antibody** |  |  |
| CD31/PECAM1 Antibody, Rabbit PAb, Antigen Affinity Purified | SinoBiological | Cat#10148-T60 |
| CD36 / SCARB3 Antibody, Rabbit PAb, Antigen Affinity Purified | SinoBiological | Cat#10752-T24 |
| Normal Rabbit IgG Control | SinoBiological | Cat# CR1-10 |
| SELP / P Selectin Antibody, Rabbit PAb | SinoBiological | Cat#13025-RP01 |
| **Chemicals, Enzymes and other reagents** |  |  |
| 1X phosphate buffer saline (PBS) | Gibco™ | Cat#20012-027 |
| AlbuMAX II™ | Gibco™ ThermoFisher Scientific | Cat#11021-037 |
| D-glucose | Sigma-Aldrich® | Cat#G7520-1KG |
| Dimethyl sulfoxide (DMSO) | Sigma-Aldrich® | Cat#D2650 |
| Fetal Bovine Serum (FBS) | ScienCell™ Research Laboratories | Cat#0025 |
| Giemsa | Sigma-Aldrich® | Cat#48900-500ML-F |
| Hypoxanthine | Sigma-Aldrich® | Cat#H9377-25G |
| IGFBP7 (25µg) | R&D Systems | Cat#1334-B7 |
| Immersion oil | Systerm | Cat#IM372-90 |
| LD Columns | Miltenvi Biotec | Cat#130-042-091 |
| L-Glutamine | Sigma-Aldrich® | Cat#G8540-100G |
| Methanol | Friendemann Schmidt | Cat#M2097-4-4001 |
| Penicillin-Streptomycin Solution | Gibco | Cat#15140122 |
| RPMI 1640 medium | Gibco™ | Cat#23400-013-1L |
| Sodium bicarbonate | Sigma-Aldrich® | Cat#S5761-500G |
| Sodium chloride | J.T.Baker | Cat#3624-69 |
| Sodium hydroxide | SYNERLAB | Cat#SY-CS10237 |
| **Software** |  |  |
| GraphPad Prism version 10.6 | GraphPad |  |
| **Others** |  |  |
| 0.5-10µl micropipette tips | Kirgen | Cat#KG5131-L |
| 1.5ml microcentrifuge tube | GSBIO | Cat#CC102-N-F |
| 10ml serological pipette | Labselect | Cat#SP-013-10 |
| 1000µl micropipette filtered tips | Axygen | Cat#TF-1000-R-S |
| 200µl micropipette filtered tips | Axygen | Cat#TF-200-L-R-S |
| 25cm^2^ cell culture flask canted neck (filter cap) | Labselect | Cat#13112A |
| 25ml serological pipette | Labselect | Cat#SP-013-25 |
| 3ml sterile transfer pipette | Biologix | Cat#30-0138A1 |
| 5ml serological pipette | Labselect | Cat# SP-013-5 |
| 500ml vacuum bottle filter | Biofil | Cat#Fpe204500 |
| 50ml centrifuge tube | Biofil | Cat#CFT01115 |
| 50mL filter syringe | Terumo | Cat#SS*50LE |
| 75cm^2^ cell culture flask canted neck (filter cap) | Labselect | Cat#13212A |
| 96-well microplates | Nunclon Surface | Cat#137101 |
| Butterfly needle | BD vacutainer | Cat#367284 |
| Cellulose acetate syringe filter  (0.22um pore size) | Bioflow Lifescience | Cat#MALCA25022 |
| Cryovial | Corning | Cat#430488 |
| Glass slide | Sail brand | Cat#7101 |
| Kimwipes | Kim Tech Science Brand | Cat#34155/34120 |
| LD columns | Miltenyi Biotec | Cat#130-042-901 |
| Minisart single-use filter unit  (Non-pyrogenic) | Sartorius stedim | Cat#S6534-FMOSK |
| Microscope coverslip 22x22mm | Mariendfeld | Cat#0101052 |
| Microscope slide with frosted side | Citoglas | Cat#P/N.0312-2101 |
| Parafilm | Bemis | Cat#PM-996 |
| QuadroMACS™ separator | Miltenyi Biotec | Cat#130-090-976 |
| Sterile Vacuum bottle filtration  (500mL 0.22µm PES filter unit) | Labselect | Cat#431097 |
| Vacutainer with clot activator (blue top) | BD | Cat# 363080 |
| Vacuitaner with heparin lithuim (green top) | BD | Cat#366480 |
| Vacuitaner plain tube (red top) | BD | Cat#367812 |

**Supplementary Table 2. Three-way ANOVA table summarizing the effects of platelets, human serum and parasite species on IRBC autoagglutination rates.**

| Source of variation | % of total variation | *p* value | DF | F (DFn, DFd) |
| --- | --- | --- | --- | --- |
| Serum factor | 16.04 | <0.0001 | 2 | F (2,21) = 35.93 |
| *P. knowlesi* vs. *P. falciparum* | 2.603 | 0.0131 | 1 | F (1,21) = 7.352 |
| Without platelets vs. with platelets | 39.37 | <0.0001 | 1 | F (1,21) = 188.8 |
| Serum factor x *P. knowlesi* vs. *P. falciparum* | 1.062 | 0.2460 | 2 | F (2,21) = 1.501 |
| Serum factor x without platelets vs. with platelets | 16.01 | <0.0001 | 2 | F (2,21) = 38.39 |
| *P. knowlesi* vs. *P. falciparum* x without platelets vs. with platelets | 1.949 | 0.0157 | 1 | F (1,21) = 6.904 |
| Serum factor x *P. knowlesi* vs. *P. falciparum* x without platelets vs. with platelets | 0.5277 | 0.4084 | 2 | F (2,21) = 0.9348 |

**Supplementary Table 3. Three-way ANOVA table summarizing the effects of platelets, human serum and parasite species on IRBC rosetting rates.**

| Source of variation | % of total variation | *p* value | DF | F (DFn, DFd) |
| --- | --- | --- | --- | --- |
| Serum factor | 52.20 | <0.0001 | 2 | F (2,21) = 61.38 |
| *P. knowlesi* vs. *P. falciparum* | 8.158 | 0.0004 | 1 | F (1,21) = 17.33 |
| Without platelets vs. with platelets | 5.720 | <0.0001 | 1 | F (1,21) = 32.87 |
| Serum factor x *P. knowlesi* vs. *P. falciparum* | 2.079 | 0.1348 | 2 | F (2,21) = 2.208 |
| Serum factor x without platelets vs. with platelets | 3.906 | 0.0005 | 2 | F (2,21) = 11.22 |
| *P. knowlesi* vs. *P. falciparum* x without platelets vs. with platelets | 0.048 | 0.6699 | 1 | F (1,21) = 0.1869 |
| Serum factor x *P. knowlesi* vs. *P. falciparum* x without platelets vs. with platelets | 0.033 | 0.9372 | 2 | F (2,21) = 0.0650 |

**Supplementary Figure 1. Rosetting rates of *P. knowlesi* (A) and *P. falciparum* (B) under IGFBP7 of different working concentrations.** Kruskal-Wallis with Dunn’s multiple comparison test was conducted. For *P. knowlesi*, significant increment in rosetting rates was found at IGFBP7 of working concentrations 50 ng/ml, 100 ng/ml and 1000 ng/ml, as compared to the rosetting rates under IGFBP7-free condition. For *P. falciparum*, significant increment of rosetting rates was found at IGFBP7 of 100 ng/ml and 1000 ng/ml. For both species, no significant difference was found between IGFBP7 concentrations of 100 ng/ml and 1000 ng/ml.
